# The timing of maternal protein degradation during mammalian preimplantation development is species-specific

**DOI:** 10.1530/REP-25-0007

**Published:** 2025-08-14

**Authors:** Veronika Kinterova, Alexandra Rosenbaum Bartkova, Shanjida Afrin, Jiri Kanka, Andrej Susor, Radek Prochazka, Tereza Toralova

**Affiliations:** ^1^ Institute of Animal Physiology and Genetics CAS, Liběchov, Czech Republic; ^2^ Constantine The Philosopher University in Nitra, Nitra, Slovakia

**Keywords:** maternal protein, protein degradation, embryonic genome activation, preimplantation development, cattle

## Abstract

**In brief:**

Proper degradation of maternally inherited proteins is a prerequisite for successful embryonic development. This study shows the species-specificity of this process.

**Abstract:**

The mechanism of targeting maternal proteins for degradation during preimplantation development is an unexplored process. Only a few proteins that need to be degraded for the proper course of the maternal-to-zygotic transition have been described in mice, and a few more in non-mammalian species. However, it is not well known whether the need for degradation is conserved across species or if it is driven in a species-specific way. Therefore, we selected six proteins that need to be degraded for the proper course of the maternal-to-zygotic transition in mice or *Xenopus*, and thoroughly characterized their expression at both the mRNA and protein level during bovine embryogenesis. Further, we analysed the protein expression in mice and pigs and compared it to bovine embryos. Thus, we provide a unique interspecies comparison of three mammalian representatives. We found that the degree of conservation between species is low and does not depend on the evolutionary relatedness of the species. This paper suggests that protein degradation during preimplantation development is controlled by a combination of species-specific factors from the embryo and the sequences of protein homologues.

## Introduction

The degradation of maternal reserves during the early phases of preimplantation development in mammals has been the subject of research interest in the last few years. While current knowledge about the degradation of maternal mRNAs is quite comprehensive, there is still not much information about maternal protein degradation. It is thought that protein degradation is not as massive a process as mRNA degradation and that it is subject to strict rules regarding the timing of degradation of individual proteins (reviewed in Toralova *et al.* (2020)). The ubiquitin-proteasome system (UPS) is traditionally considered to be the main player in maternal protein degradation (Suzumori *et al.* 2003, DeRenzo & Seydoux 2004, Mtango & Latham 2007, Verlhac *et al.* 2010). Ubiquitin has been shown to be a marker of developing blastocysts (Katz-Jaffe *et al.* 2006), and the ubiquitin-proteasome pathway is essential for many cellular processes such as the cell cycle or the regulation of transcription (Yao & Ndoja 2012). Several E3 ubiquitin ligases (RNF114, SCF complex DCAF13 and others) are involved in maternal protein degradation (Benesova *et al.* 2016, Yang *et al.* 2017, Kinterova *et al.* 2019, Zhou *et al.* 2021, Kinterová *et al.* 2022, da Silva *et al.* 2023). Moreover, autophagy and clathrin-mediated endocytosis also play an unquestionable and irreplaceable role in protein degradation during preimplantation development (Tsukamoto *et al.* 2008, Morita *et al.* 2021).

It seems that only a small proportion of maternal proteins are degraded during the maternal-to-zygotic transition (MZT, S Box 1 (see section on Supplementary materials given at the end of the article)) (Cao *et al.* 2020). To date, the degradation of only a few such proteins has been identified as crucial for the normal course of embryonic genome activation (EGA) in mammals (Yang *et al.* 2017, Higuchi *et al.* 2019, Zhou *et al.* 2021, Li *et al.* 2022), and a few more in lower organisms – *Drosophila* and *Xenopus* (Shimuta *et al.* 2002, Kanemori *et al.* 2005, Collart *et al.* 2013, 2017). Nevertheless, identifying proteins that need to be degraded for the proper course of preimplantation development is crucial for characterizing the way in which proteins are targeted for degradation during this developmental period, as species-specific and embryo-specific pathways seem to be involved in this process. The proteins targeted for degradation usually ensure the high speed of the cell cycle and/or chromatin silencing during the early stages of embryogenesis, but proteins degraded in one species are often preserved in another, and vice versa.

The aim of this article is to characterize the expression profiles of bovine genes whose proteins need to be degraded for the proper course of EGA/MBT (midblastula transition) in other species. The results can thus serve as a basis for the analysis of maternal protein processing during preimplantation development in cattle and for characterizing the mechanism by which maternal proteins are processed during preimplantation development.

## Materials and methods

### Bovine *in vitro* fertilization (IVF) and embryo culture

Bovine embryos were obtained after the *in vitro* maturation of oocytes and their subsequent IVF and *in vitro* culture. Abattoir-derived ovaries from cows and heifers were collected and transported in thermo-containers. Cumulus-oocyte complexes (COCs) were isolated, matured and fertilized, and the embryos were cultured and fixed as described in Benesova *et al.* (2017).

### Pig oocyte collection, parthenogenetic activation and embryo culture

Pig ovaries were collected from prepubertal gilts at a local abattoir. COCs were isolated and cultured in FLI medium and consequently parthenogenetically activated as described in Rosenbaum Bartkova *et al.* (2024).

A group of 50 putative parthenotes was washed twice in porcine zygote medium (PZM) 3 and cultivated for 24 h (2-cell), 30 h (early 4-cell), 48 h (late 4-cell), 72 h (8-cell), 96 h (morula) and 144 h (blastocyst) in 500 μL of PZM 3 medium at 38.5°C under a 5% CO_2_ atmosphere (Yoshioka *et al.* 2002).

### Mouse embryo collection and culture

Female mice were stimulated with 5 IU (international unit) of pregnant mare serum gonadotrophin (PMSG; Folligon; Merck Animal Health) per mouse. The PMSG-stimulated mice were then injected with 5 IU of hCG (human chorionic gonadotrophin) before mating overnight with males of the same strain. After 16 hours, zygotes were recovered from the oviducts and cultured in M16 medium. After 4 hours, zygotes were transferred to KSOM media (MR-106-D, EmbryoMax, Merck, Germany) covered with oil (Ovoil, 10029, Vitrolife, Sweden) and cultured in a humidified atmosphere with 5% CO2 at 37.5°C. To detect the timing of EGA in our mouse strain (ICR mouse), we explored the activity of RNA polymerase II using immunofluorescence staining of its phosphorylated form and found transcription activation already at late zygotes (Fig. S1). Zygotes (pre-EGA stage) were collected at 20 h, 2c at 34 h, 4c at 60 h, 8c at 70 h, morula at 93 h and blastocyst at 108 h after hCG injection. All animal experiments were performed according to the guidelines and protocols approved by the Laboratory of Biochemistry and Molecular Biology of Germ Cells at the Institute of Animal Physiology and Genetics in the Czech Republic (‘Mouse Oocyte Isolation, Cultivation and RNA Microinjection’, b.r.). All animal experiments were conducted in accordance with Act No. 246/1992 on the Protection of Animals from Cruelty, issued by the Ministry of Agriculture within the framework of the experimental project No. 67756/2020MZE-18134.

### Western blot and immunoprecipitation

Unless otherwise indicated, all chemicals were purchased from Thermo Fisher. Embryos and oocytes (cattle and pigs: 20 per extract; mice: 40 per extract) were lysed in 9.5 μL of a mixture of NuPAGE LDS Sample Buffer (4X) and NuPAGE Sample Reducing Agent and boiled. The mixture was subjected to NuPAGE 4–12% Bis-Tris Gel. Proteins were transferred from gels to an Immobilon P membrane (Millipore Biosciences, USA) using a semidry blotting system, Trans-Blot Turbo Transfer System (Bio-Rad, Czech Republic). The blocking of the membrane was performed in Chemi Blot Blocking Buffer (Azure Biosystems, USA) or 3% BSA (bovine serum albumin) in TBS-Tween buffer (TBS-T, 20 mM Tris, pH 7.4, 137 mM NaCl and 0.5% Tween 20), and incubated overnight with a primary antibody in the corresponding blocking solution (Table S1; interspecies protein homology relevant to antibody cross-reactivity is shown in Fig. S2) and subsequently with HRP-conjugated secondary antibody (1:7,500; Jackson ImmunoResearch, USA) in the corresponding blocking solution for 1 h. Proteins were visualized using ECL (Amersham). The data were processed using Azure 600 (Azure Biosystems). The quantification of band intensities was performed using Fiji Software by Gel Analysis tool.

The immunoprecipitation was performed using Dynabeads Co-Immunoprecipitation Kit according to the manufacturer´s instructions. Briefly, for binding of the ubiquitinated proteins to the beads, 10 μg of anti-ubiquitin antibody was used. The mixture was incubated rotating for 24 h at 37°C, washed and diluted 100 μL per 1 mg of beads. The beads were stored at 4°C until use. Fifty embryos per sample were used. 200 μL of extraction buffer (5x IP Buffer provided in the kit, diluted fivefold with RNase-free H_2_O, 100 mM NaCl and 2 μL of Halt protease) was added to the sample, centrifuged, added to the prepared Dynabeads and incubated rotating for 30 min at 4°C. After washing using the provided solutions, 4 μL of SDS reducing buffer and 1.5 μL of sample reducing agent were added and the sample was subjected to NuPAGE 4–12% Bis-Tris Gel and performed as indicated above.

### Immunofluorescence

Oocytes or embryos were treated as in (Benesova *et al.* 2016). Samples were blocked with 2% normal goat serum (NGS; Millipore Biosciences) or PBS supplemented with 5% BSA for 1 h and incubated with appropriate primary (see Table S1) and secondary antibody. Afterwards, the nuclei were stained and embryos were mounted in SlowFade Diamond Antifade Mountant with DAPI (4′,6-diamidine-2′-phenylindole dihydrochloride, Invitrogen, Thermo Fisher Scientific, Czech Republic). Controls of immunostaining specificity were carried out by omitting the primary antibody.

The samples were examined using Leica TCS SP5 (Leica Microsystems AG, Germany). The images were processed using Fiji software, and Fiji was also used for quantitative analysis of protein localization differences. The relative fluorescence intensity in several whole nuclei (or in DNA in the case of MII oocytes) and in three defined cytoplasmic regions of interest (ROIs in μm^2^) per oocyte or embryo were measured. For each developmental stage, between five and ten oocytes/embryos were analysed. Fluorescence values were normalized to the total fluorescence intensity of each oocyte/embryo, and the resulting relative intensities are shown as line graphs to demonstrate the subcellular protein distribution changes.

### Quantification of mRNA expression

Oocytes and embryos were washed using PBS and stored dry and deep-frozen at −80°C. RNA was extracted using RNeasy Plus Mini Kit (Qiagen, Germany) according to the manufacturer’s instructions.

The expression of mRNA was measured by quantitative RT-PCR (qRT-PCR), and the reaction was performed as in (Benesova *et al.* 2016) using fluorescence EvaGreen Dye. Primers used are listed in Table S2. The 10 μL reaction mixture contained 2 μL 5x reaction buffer, 0.4 μL dNTP mix (10 nM stock), 0.2 μL forward and reverse primers (20 nM stock), 0.4 μL enzyme mix, 0.4 μL EvaGreen (Biotium, USA), 2 μL RNA and nuclease-free water. Products were verified using melting analysis and gel electrophoresis on 1.5% agarose gel with MidoriGreen Direct (Nippon Genetics, Japan). Gene expression was analysed after normalization to the reference gene H3F3A (H3 histone, family 3A) as the internal control gene using Corbett Research comparative analysis software.

The expression of *Tab1* mRNA was measured by two-step real-time qPCR. For reverse transcription, the Maxima H Minus First Strand cDNA Synthesis Kit (Thermo Scientific) was used according to the manufacturer’s instructions under the following conditions: 10 min at 25°C, 30 min at 50°C and 5 min at 85°C. Afterwards, cDNA was used for qPCR using Phusion High-Fidelity PCR Kit (Thermo Scientific) in RotorGene 3000 cycler in a 20 μL reaction mixture containing 4 μL of 5x HF buffer, 0.4 μL dNTP mix, 0.4 μL forward and reverse primers, 0.2 μL enzyme, 0.8 μL EvaGreen, 1 μL cDNA and nuclease-free water. Further analysis was performed as indicated above.

### Statistical analysis

The data were analysed using SigmaStat 3.0 software (Jandel Scientific, USA). For multiple alignments, the Holm-Sidak method was used; for pairwise alignments, the pairwise *t*-test was used. *P* ≤ 0.05 was considered statistically significant.

## Results

### The timing of TOPBP1 protein degradation correlates with mRNA depletion in bovine embryos

**Figure 1:**
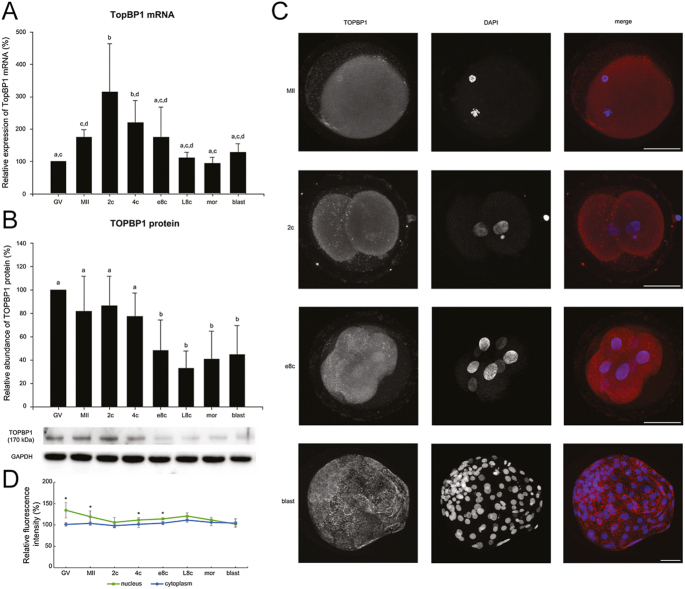
Temporal expression pattern of *TOPBP1* gene in bovine oocytes and embryos. (A) Relative levels of *TOPBP1* mRNA (qRT-PCR expression analysis). (B) Relative levels of TOPBP1 protein (Western blot analysis). The representative Western blot picture and the loading control using anti-GAPDH antibody are shown below the graph. (C) Localization of TOPBP1 protein (confocal laser scanning microscopy). Representative images of each type of localization are shown. Scale bars: 50 μm, DNA (DAPI): blue, TOPBP1: red. (D) Relative nuclear and cytoplasmic localization of TOPBP1 during oocyte maturation and early embryonic development. Bars in (A) and (B) show mean ± S.D., the expression level of GV oocytes was considered 100%. ^a, b, c, d^ values with different superscripts indicate statistical significance (*P* < 0.05; (Holm–Sidak test)). The line graph in (D) shows the relative fluorescence intensity (%) of the protein, shown as mean ± S.D. Statistical significance (*P* < 0.05; *t*-test) between nuclear and cytoplasmic localization within each developmental stage is indicated by an asterisk (*). All the experiments were repeated at least three times. GV, germinal vesicle stage oocyte; MII, metaphase II stage oocyte; 2c, 2-cell stage embryo; 4c, 4-cell stage embryo; e8c, early 8-cell stage embryo; L8c, late 8-cell stage embryo; mor, morula; blast, blastocyst.

Topoisomerase 2 β-binding protein 1 (TOPBP1) is a DNA replication factor whose degradation regulates cell-cycle lengthening during MBT in Xenopus (Collart *et al.* 2013). Therefore, we wanted to know whether its degradation is a common feature during EGA/MBT. We found a statistically significant decrease in TOPBP1 protein levels in bovine embryonic stages from e8c onwards compared to the preceding stages and oocytes (*P* < 0.05) (Fig. 1B). Protein expression in embryonic stages correlated with mRNA level, as there was a significant increase in *TOPBP1* mRNA at the 2c stage (*P* < 0.03; 2c vs MII), and the level gradually decreased thereafter (*P* < 0.05 2c vs e8c; *P* < 0.005 vs L8c, morula and blastocyst) (Fig. 1A).

The TOPBP1 protein was diffusely localized to both nuclei and cytoplasm. Nuclear staining was most significant at 4c and e8c stages (Fig. 1D). At the blastocyst stage, TOPBP1 formed protein aggregates. In the GV and MII oocyte, the protein was localized to the chromosomes (Fig. 1C and D).

The level of TOPBP1 protein decreased significantly after fertilization (*P* < 0.001 MII vs 2c), and remained approximately the same until the blastocyst stage in pig embryos. In mouse embryos, a clear but non-significant decrease in TOPBP1 levels was observed after fertilization, which then decreased slightly at the morula stage (*P* < 0.05 morula vs MII), and even more significantly at the blastocyst stage (*P* < 0.05 blastocyst vs MII) (Fig. 7A).

### DBF4B protein is not detectable in mouse embryos

We further wanted to know whether the decrease in protein abundance during EGA is conserved across species for some of the other replication factors studied in *Xenopus*. Thus, we selected the dumbbell-forming 4B (DBF4B).

**Figure 2:**
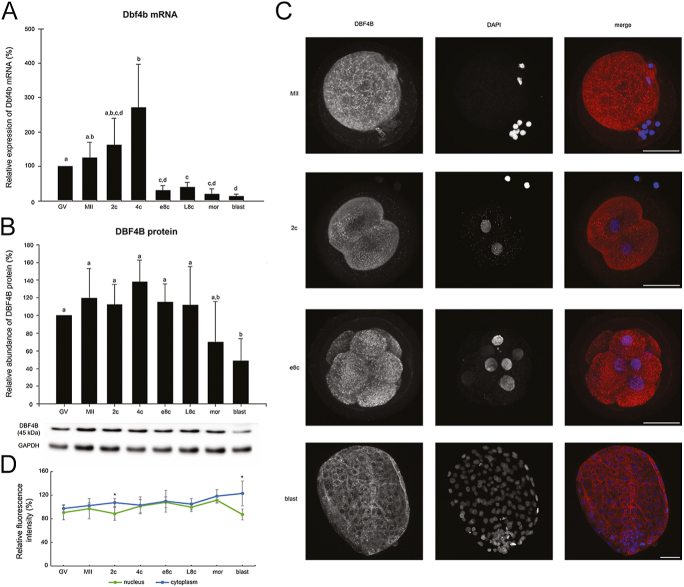
Temporal expression pattern of *DBF4B* gene in bovine oocytes and embryos. (A) Relative levels of *DBF4B* mRNA (qRT-PCR expression analysis). (B) Relative levels of DBF4B protein (western blot analysis). The representative western blot picture and the loading control using anti-GAPDH antibody are shown below the graph. (C) Localization of DBF4B protein (confocal laser scanning microscopy). Representative images of each type of localization are shown. Scale bars: 50 μm, DNA (DAPI): blue, DBF4B: red. (D) Relative nuclear and cytoplasmic localization of DBF4B during oocyte maturation and early embryonic development. Bars in (A) and (B) show mean ± S.D., the expression level of GV oocytes was considered 100%. ^a, b, c, d^ values with different superscripts indicate statistical significance (*P* < 0.05; Holm–Sidak test). The line graph in (D) shows the relative fluorescence intensity (%) of the protein, shown as mean ± S.D. Statistical significance (*P* < 0.05; *t*-test) between nuclear and cytoplasmic localization within each developmental stage is indicated by an asterisk (*). All the experiments were repeated at least three times. GV, germinal vesicle stage oocyte; MII, metaphase II stage oocyte; 2c, 2-cell stage embryo; 4c, 4-cell stage embryo; e8c, early 8-cell stage embryo; L8c, late 8-cell stage embryo; mor, morula; blast, blastocyst.

The level of DBF4B protein was quite stable from the GV oocyte to the late 8-cell stage in cattle, i.e. beyond the EGA stage. Thereafter, its amount decreased at the morula and blastocyst stages, but it was only at the latter stage that the decrease was statistically significant compared to the stages from the GV oocyte to the late 8-cell stage (*P* < 0.05) (Fig. 2B). The protein was diffusely expressed throughout the whole embryo, with less pronounced staining in the nuclei. Significant decrease of nuclear staining was observed, particularly at the 2-cell stage. From 4c to L8c stage, the nuclear signal was higher and then, in morula, differences in nuclear and cytoplasmic signal started to increase again, followed by a significant reduction of nuclear localization at the blastocyst (Fig. 2C and D). The decrease in mRNA occurred at the early 8-cell stage (*P* = 0.009 4c vs e8c), and remained low until the blastocyst stage (Fig. 2A).

In mouse oocytes and embryos, the DBF4B protein was not detectable. In pig embryos, the expression profile was similar to that in cattle (Fig. 7B).

### A high amount of CDC25A protein is preserved until later stages of preimplantation development in mice and pigs

Cell division cycle 25A (CDC25A) is a phosphatase responsible for positive regulation of the cell-cycle, and its degradation is crucial for cell cycle lengthening after MBT in *Xenopus* and *Drosophila* (Shimuta *et al.* 2002, Kanemori *et al.* 2005, Farrell *et al.* 2012). Thus, we wanted to know whether such degradation also occurs in mammals.

**Figure 3:**
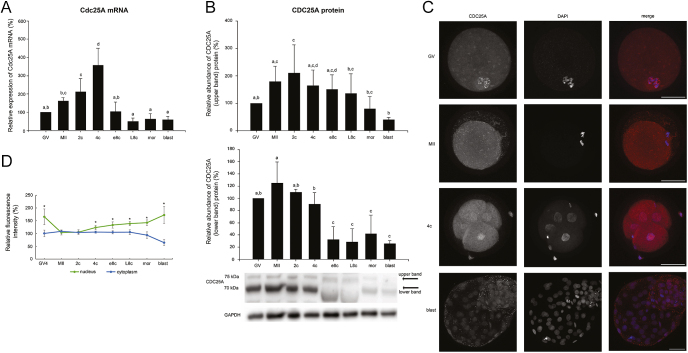
Temporal expression pattern of *CDC25A* gene in bovine oocytes and embryos. (A) Relative levels of *CDC25A* mRNA (qRT-PCR expression analysis). (B) Relative levels of the upper and lower bands of CDC25A protein (western blot analysis). The representative western blot picture and the loading control using anti-GAPDH antibody are shown below the graph. (C) Localization of CDC25A protein (confocal laser scanning microscopy). Representative images of each type of localization are shown. Scale bars: 50 μm, DNA (DAPI): blue, CDC25A: red. (D) Relative nuclear and cytoplasmic localization of CDC25A during oocyte maturation and early embryonic development. Because of differences in localization at GV oocytes, only oocytes in GV4 stage were selected for analysis. Bars in (A) and (B) show mean ± S.D., the expression level of GV oocytes was considered 100%. ^a, b, c, d^ values with different superscripts indicate statistical significance (*P* < 0.05; Holm–Sidak test). The line graph in (D) shows the relative fluorescence intensity (%) of the protein, shown as mean ± S.D. Statistical significance (*P* < 0.05; *t*-test) between nuclear and cytoplasmic localization within each developmental stage is indicated by an asterisk (*). All the experiments were repeated at least three times. GV, germinal vesicle stage oocyte; MII, metaphase II stage oocyte; 2c, 2-cell stage embryo; 4c, 4-cell stage embryo; e8c, early 8-cell stage embryo; L8c, late 8-cell stage embryo; mor, morula; blast, blastocyst.

A double band of an approximate molecular weight of 70 and 75 kDa was detected in bovine embryos. In both 8-cell stages, the lower band was shifted to a lower molecular weight (approx. 65–67 kDa). In blastocysts, the entire double band was shifted down by 2–3 kDa, and the signal was very weak. The intensity of the higher band was highest at 2c, and gradually decreased thereafter until the blastocyst stage. The decrease was statistically significant at the morula and blastocyst stages (*P* < 0.05 morula vs MII and 2c; blastocyst vs MII, 2c, 4c, e8c). The intensity of the lower band decreased significantly at the e8c stage and all subsequent stages compared to oocytes, 2c and 4c (*P* ≤ 0.001 in each case) (Fig. 3B). The level of mRNA corresponded to the expression profile of the lower protein band, as the level of *CDC25A* mRNA decreased rapidly at the e8c stage in comparison to 4c stage and remained low until the blastocyst stage (*P* < 0.01) (Fig. 3A).

In oocytes, the localization of the CDC25A protein was dependent on the meiotic phase. In the earlier stages, the protein was colocalized with chromosomes; during the GV4 stage, the localization changed to a diffuse distribution throughout the nucleoplasm. In the MII oocyte and 2c, the protein was diffusely localized throughout the cytoplasm. From the 4c to the morula stage, the intensity of nuclear staining significantly increased, but cytoplasmic staining was still considerable. At the blastocyst stage, the protein was mainly localized to the nucleoplasm and only to a small extent to the cytoplasm (Fig. 3D). In cumulus cells, the protein was present almost exclusively in nuclei at oocyte stages, but shifted to the cytoplasm at embryonic stages (Fig. 3C).

Interestingly, the high levels of CDC25A persisted beyond EGA in both pig and mouse embryos. In pig embryos, high levels of CDC25A protein were detected up to the morula stage and decreased significantly at the blastocyst stage (*P* ≤ 0.05 blastocyst vs all preceding stages). In mouse embryos, the CDC25A level remained high until 8c. At the morula stage, it started to decrease (*P* < 0.05 morula vs GV, MII, 2c, 4c), and decreased dramatically at the blastocyst stage (*P* < 0.001 blastocyst vs all preceding stages) (Fig. 7C).

### Neither the *CBX5* mRNA nor the CBX5 protein is degraded during bovine preimplantation development

**Figure 4:**
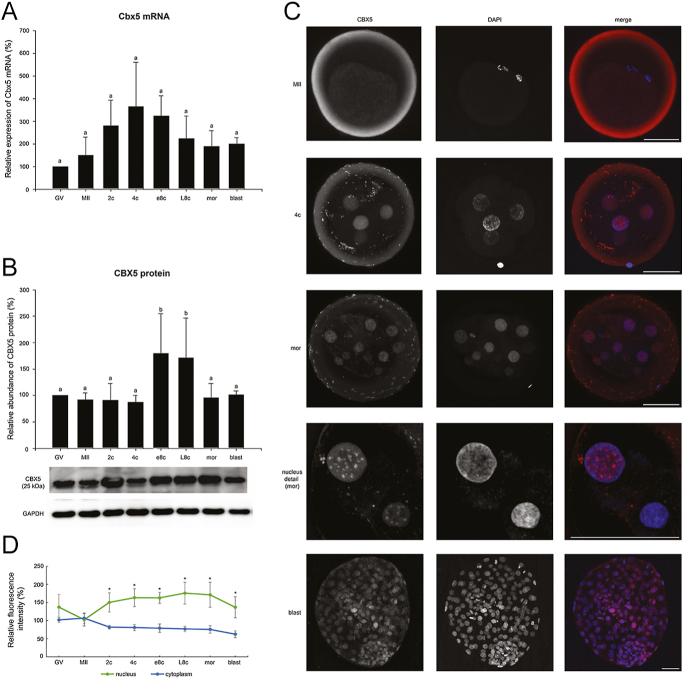
Temporal expression pattern of *CBX5* gene in bovine oocytes and embryos. (A) Relative levels of *CBX5* mRNA (qRT-PCR expression analysis). (B) Relative levels of CBX5 protein (western blot analysis). The representative western blot picture and the loading control using anti-GAPDH antibody are shown below the graph. (C) Localization of CBX5 protein (confocal laser scanning microscopy). Representative images of each type of localization and nuclear detail are shown. Scale bars: 50 μm, DNA (DAPI): blue, CBX5: red. (D) Relative nuclear and cytoplasmic localization of CBX5 during oocyte maturation and early embryonic development. Bars in (A) and (B) show mean ± S.D., the expression level of GV oocytes was considered 100%. ^a, b^ values with different superscripts indicate statistical significance (*P* < 0.05; Holm–Sidak test). The line graph in (D) shows the relative fluorescence intensity (%) of the protein, shown as mean ± S.D. Statistical significance (*P* < 0.05; *t*-test) between nuclear and cytoplasmic localization within each developmental stage is indicated by an asterisk (*). All the experiments were repeated at least three times. GV, germinal vesicle stage oocyte; MII, metaphase II stage oocyte; 2c, 2-cell stage embryo; 4c, 4-cell stage embryo; e8c, early 8-cell stage embryo; L8c, late 8-cell stage embryo; mor, morula; blast, blastocyst.

Chromobox 5 (CBX5 or heterochromatin protein 1 alpha, HP1α) is a member of the chromobox protein family and works as a methyl reader of methylated H3K9 (van Wijnen *et al.* 2021). In mouse embryos, CBX5 protein overexpression leads to an improper course of major EGA (Zhou *et al.* 2021). Therefore, we wanted to describe the CBX5 expression profile in another mammalian species. Nevertheless, we detected no decrease in protein level around EGA. On the contrary, there was a statistically significant increase at e8c and L8c (*P* < 0.05 vs all other stages) (Fig. 4B). Similarly, no statistically significant differences were found in mRNA level during bovine preimplantation development (*P* < 0.05 in each case) (Fig. 4A).

The protein was diffusely localized to the nuclei in early embryonic stages, and its nuclear localization significantly increased from the 2c stage onwards (Fig. 4D). From e8c, it reflected the establishment of chromocentres. The chromocentre-specific localization was most evident at the morula stage (Fig. 4C).

In all the tested species, the expected band at 25 kDa was present, but in pigs and especially in mice, this band was very weak and the band between 30 and 40 kDa was dominant (Fig. S3). Since we wanted to describe the timing of total protein elimination, we compared the intensity of the dominant bands of each species, as this represents the majority of the protein in the embryo. In contrast to cattle, a mild decrease in the protein level was found around EGA in both mice and pigs (Fig. 7D).

### The timing of PIASY degradation is conserved across species

**Figure 5:**
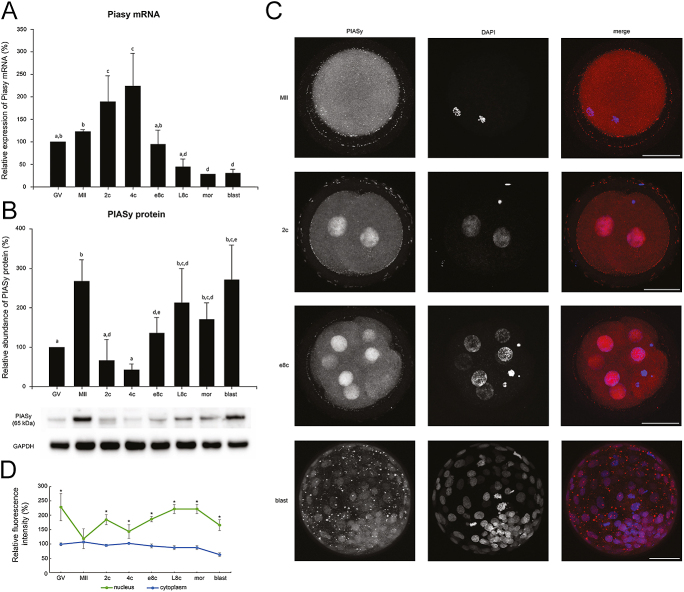
Temporal expression pattern of *PIASY* gene in bovine oocytes and embryos. (A) Relative levels of *PIASY* mRNA (qRT-PCR expression analysis). (B) Relative levels of PIASY protein (western blot analysis). The representative western blot picture and the loading control using anti-GAPDH antibody are shown below the graph. (C) Localization of PIASY protein (confocal laser scanning microscopy). Representative images of each type of localization are shown. Scale bars: 50 μm, DNA (DAPI): blue, PIASY: red. (D) Relative nuclear and cytoplasmic localization of PIASY during oocyte maturation and early embryonic development. Bars in (A) and (B) show mean ± S.D., the expression level of GV oocytes was considered 100%. ^a, b, c, d, e^ values with different superscripts indicate statistical significance (*P* < 0.05; Holm–Sidak test). The line graph in (D) shows the relative fluorescence intensity (%) of the protein, shown as mean ± S.D. Statistical significance (*P* < 0.05; *t*-test) between nuclear and cytoplasmic localization within each developmental stage is indicated by an asterisk (*). All the experiments were repeated at least three times. GV, germinal vesicle stage oocyte; MII, metaphase II stage oocyte; 2c, 2-cell stage embryo; 4c, 4-cell stage embryo; e8c, early 8-cell stage embryo; L8c, late 8-cell stage embryo; mor, morula; blast, blastocyst.

Protein Inhibitor of Activated STAT 4 (PIASY/ PIAS4) is a SUMO (small ubiquitin-like modifier) E3 ligase which is involved in the regulation of several important signalling pathways (Albor *et al.* 2006, Sharrocks 2006). It was found to be degraded after fertilization in mice, and this degradation is necessary for the switch between the minor and major wave of EGA (Higuchi *et al.* 2019). In bovine embryos, we found a statistically significant decrease in protein level in the 2-cell stage and 4-cell stage (*P* < 0.001 2c and 4c vs MII) compared to MII stage oocytes. From the early 8-cell stage to the blastocyst stage, the level of PIASY protein increased significantly compared to the 4-cell stage (*P* < 0.05) (Fig. 5B). The mRNA level exhibited the opposite expression profile. It gradually increased from the GV oocyte to the 4-cell stage, and decreased significantly at the e8c (*P* ≤ 0.005 e8c vs 2c and 4c), and even more significantly in the following stages (L8c vs MII, 2c and 4c *P* ≤ 0.016; morula and blastocyst *P* < 0.05 vs GV and e8c, *P* ≤ 0.006 vs MII, 2c and 4c) (Fig. 5A).

The protein exhibited strong nuclear staining in all developmental stages. In the oocyte stages, the protein was diffusely expressed in the cytoplasm and colocalized with the DNA, especially in the GV stage. In all early embryonic stages, the signal in nuclei was significantly higher than in the cytoplasm (Fig. 5D). At stages 2c, 4c, e8c, and in the cumulus cells, the protein was also diffusely expressed in the cytoplasm in addition to nuclear staining. From L8c onwards, the protein began to form clusters in the cytoplasm, and only numerous clusters instead of diffuse staining were found at the blastocyst stage in trophectoderm (TE) cells. In the inner cell mass (ICM), the staining was also diffusely expressed in the cytoplasm. From the 8-cell stage, the staining was absent from nucleoli (Fig. 5C).

The trend of protein abundance in pigs and mice was similar to that in cattle. The decrease in protein level was statistically significant in the 2c stage compared to MII stage oocytes in both pigs and mice (*P* < 0.05). In bovine and mouse embryos, we found an increase in protein level that started at the EGA stage (mouse) or before the EGA stage (4c, bovine embryos). In pigs, the protein level remained low throughout the whole of preimplantation development (Fig. 7E).

### The degradation of TAB1 occurs after fertilization in mice and pigs and before the EGA stage in mice and cattle

TGF-beta activated kinase 1 binding protein 1 (TAB1) is a regulator of Nuclear Factor kappa-light-chain-enhancer of activated B cells (NF-κB) and mitogen-activated protein kinase (MAPK) pathways. Its degradation during mouse EGA is needed for NF-κB activation and consequent EGA initiation (Yang *et al.* 2017). Thus, we wanted to see whether a similar expression profile is also typical for other mammalian embryos. In bovine embryos, we detected a double band around 50 kDa, a band close to 60 kDa, a weak band around 75 kDa (Fig. S4), and an uncharacterized band at 40 kDa (presumably a truncated splicing variant). At the early and late 8-cell stage, a band with a molecular weight of about 65 kDa appeared, which likely represents a stage-specific post-translational modification (PTM). This band does not appear in any other stages of bovine oocytes/embryos from GV oocytes to blastocysts.

**Figure 6:**
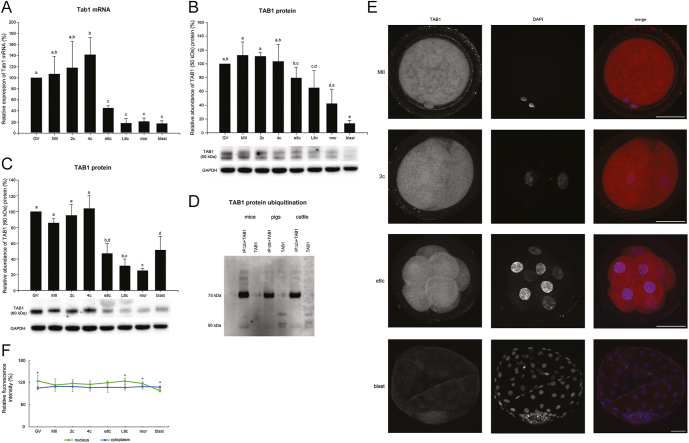
Temporal expression pattern of *TAB1* gene in bovine oocytes and embryos. (A) Relative levels of *TAB1* mRNA (qRT-PCR expression analysis). (B) Relative levels of TAB1 protein (50 kDa) (western blot analysis). The representative western blot picture and the loading control using anti-GAPDH antibody are shown below the graph. (C) Relative levels of TAB1 protein (60 kDa) (western blot analysis). The representative western blot picture and the loading control using anti-GAPDH antibody are shown below the graph. (D) Detection of TAB1 ubiquitination. The ubiquitylated proteins were immunoprecipitated by anti-ubiquitin antibody and further analysed by western blotting using anti-TAB1 antibody. The representative image shows ubiquitylated TAB1 protein (IP:Ub + TAB1) and total TAB1 protein (TAB1) in mouse and pig MII oocytes and bovine 8c-stage embryos (8c in cattle showed additional higher-molecular-weight bands missing in other stages). (E) Localization of TAB1. Representative images of each type of localization are shown. (F) Relative nuclear and cytoplasmic localization of TAB1 during oocyte maturation and early embryonic development. Bars in (A), (B) and (C) show mean ± S.D., the expression level of GV oocytes was considered 100%. ^a, b, c, d, e^ values with different superscripts indicate statistical significance (*P* < 0.05; Holm–Sidak test). The line graph in (F) shows the relative fluorescence intensity (%) of the protein, shown as mean ± S.D. Statistical significance (*P* < 0.05; *t*-test) between nuclear and cytoplasmic localization within each developmental stage is indicated by an asterisk (*). All the experiments were repeated at least three times. GV, germinal vesicle stage oocyte; MII, metaphase II stage oocyte; 2c, 2-cell stage embryo; 4c, 4-cell stage embryo; e8c, early 8-cell stage embryo; L8c, late 8-cell stage embryo; mor, morula; blast, blastocyst.

The level of the protein double band at 50 kDa was quite stable from the GV oocyte to 4c, and gradually decreased from e8c (*P* < 0.05 e8c vs 2c), with the most significant decline at the blastocyst stage (*P* ≤ 0.002 blastocyst vs all preceding stages except morula) (Fig. 6B). The level of the 60 kDa band was high from the GV stage oocyte to the 4c stage embryo, decreased sharply at e8c (*P* < 0.001 e8c vs GV, MII, 2c, 4c), continued decreasing until the morula stage (*P* < 0.001 L8c and morula vs GV, MII, 2c, 4c), and increased slightly at the blastocyst stage (*P* = 0.012 blastocyst vs morula) (Fig. 6C). The band at 75 kDa represents a ubiquitylated form of TAB1, as was shown by ubiquitin-TAB1 immunoprecipitation (Fig. 6D). The level of mRNA was also low at the later stages of development. It was high from the GV stage oocyte to 4c, and decreased sharply thereafter (*P* ≤ 0.011 e8c vs GV, MII, 2c and 4c) (Fig. 6A).

Protein was diffusely expressed in both the cytoplasm and nuclei in oocytes, cumulus cells and embryos, with significantly increased nuclear staining in GV oocyte, L8c and morula. Contrarily, the protein exhibited only minimal nuclear expression at the blastocyst stage (Fig. 6E and F).

**Figure 7:**
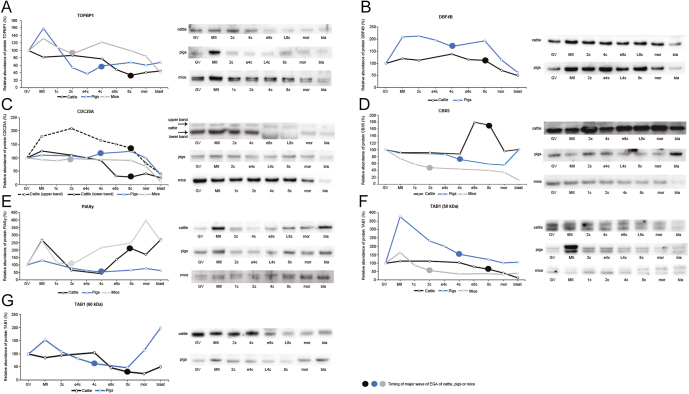
Temporal expression patterns of individual proteins in various mammalian species. (A) TOPBP1; (B) DBF4B; (C) CDC25A; (D) CBX5; (E) PIASY; (F) TAB1 (50 kDa); and (G) TAB1 (60 kDa). The timing of the major wave of EGA is marked by large circles in colour corresponding to the data series of each species. Timing of EGA: mice: early 2-cell stage (Aoki *et al.* 1997); pigs: 4-cell stage (Arrell *et al.* 1991); cattle: late 8-cell stage (Frei *et al.* 1989, Kanka *et al.* 2012). The representative Western blot pictures for each species are shown alongside the graphs. GV, germinal vesicle stage oocyte; MII, metaphase II stage oocyte; 1c, zygote; 2c, 2-cell stage embryo; e4c, early 4-cell stage embryo; 4c, 4-cell stage embryo; e8c, early 8-cell stage embryo; 8c, 8-cell stage embryo; mor, morula; blast, blastocyst.

In pig oocytes and embryos, bands of the same size as in cattle appeared, and only several additional bands between 70 and 120 kDa were noticeable. In mice, TAB1 was primarily detected as a double band at approximately 55–60 kDa. The molecular weight of the higher band increased at the morula, and especially at the blastocyst stage. Two weak bands at around 48 kDa and several weaker bands of higher molecular weights (70 kDa, 100 kDa) appeared as well. Bands at approximately 50 kDa in cattle and pigs and 55 kDa in mice were chosen for the comparison of expression profiles, as they represent the unmodified form of the protein (Fig. S4). While the level of the 50 kDa band was stable until 4c in bovine embryos, its level gradually decreased from the MII oocyte to blastocyst stage in pig embryos. In mice, the level of protein decreased from the MII oocyte to the 4c stage and remained low until the blastocyst stage (*P* < 0.05 MII vs all embryonic stages). This means that in pig and mouse embryos, TAB1 degradation started after fertilization, while in bovine embryos, the degradation is less and occurs shortly before EGA (Fig. 7F). The expression profile of the TAB1 band at approximately 60 kDa was compared in pigs and cattle, as no band at this height was detected in mice. In pig embryos, it gradually decreased from the MII stage to the 8c stage (*P* < 0.001 MII vs e4c, L4c, 8c), which means that degradation of the protein started after fertilization, much earlier than in bovine embryos. Thereafter, its level started to increase at the morula stage and increased significantly at the blastocyst stage (*P* < 0.05 blastocyst vs all preceding embryonic stages). The increase at the morula and blastocyst stage was much more distinct in pig than in bovine embryos (Fig. 7G).

## Discussion

Based on current knowledge, the degradation of maternal proteins seems to be species-specific in mammals. For example, the degradation of H1FOO (oocyte and early embryonic variant of linker histone H1), which is one of the most abundantly expressed genes in oocytes (Gad *et al.* 2020), is needed for the 8- to 16-cell stage transition and TE differentiation in cattle, and the overexpression of bovine H1FOO causes defects in blastocyst development in both bovine and mouse embryos. On the other hand, the overexpression of mouse H1FOO does not impair development (Li *et al.* 2022).

**Figure 8:**
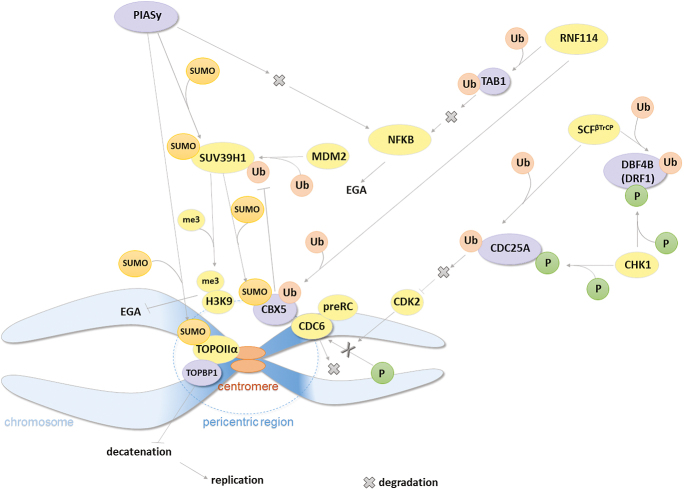
The interplay of the studied proteins in the regulation of the cell cycle. The studied proteins are coloured in mauve. βTrCP, β-transducin repeat-containing protein; CBX5, chromobox 5; CDC6, cell division cycle 6; CDK2, cyclin-dependent kinase 2; CHK1, checkpoint kinase 1; DBF4B, dumbbell forming 4B; DRF1, DBF4-related factor 1; EGA, embryonic genome activation; H3K9, histone H3 lysine 9; MDM2, mouse double minute 2 homologue; me3, trimethylation; NFκB, nuclear factor kappa-light-chain-enhancer of activated B cells; P, phosphorylation; PIASy, protein inhibitor of activated STAT 4; preRC, prereplication complex; RNF114, ring finger protein 114; SCF, SKP1/Cullin1/F-box–protein complex; SUMO, small ubiquitin-like modifier; SUV39H1, suppressor of variegation 3–9 homologue 1; TAB1, TGF-beta activated kinase 1 binding protein 1; TOPBP1, topoisomerase 2 β-binding protein 1; TOPOIIα, topoisomerase 2 α isotype; Ub, ubiquitination. (Azuma *et al.* 2005, Albor *et al.* 2006, Díaz-Martínez *et al.* 2006, Martin *et al.* 2006, Collart *et al.* 2013, 2017, Maison *et al.* 2016, Raurell-Vila *et al.* 2017).

To determine whether such an interspecies discrepancy is a common phenomenon, we characterized protein and mRNA expression of bovine homologues of proteins whose degradation is needed for the proper course of the maternal-to-zygotic transition in mice or *Xenopus*. Namely, CDC25A, DBF4B, TOPBP1, CBX5, PIASY and TAB1 were characterized. The role of all studied proteins during preimplantation development is shown in Fig. 8. Further, we characterized the protein expression profiles in mouse and pig embryos to see whether such processing is also typical for other mammals. The preferably used way of *in vitro* preparation of embryos was used in each model organism. Such a comparison of several mammalian species in one study is quite unique.

**Figure 9:**
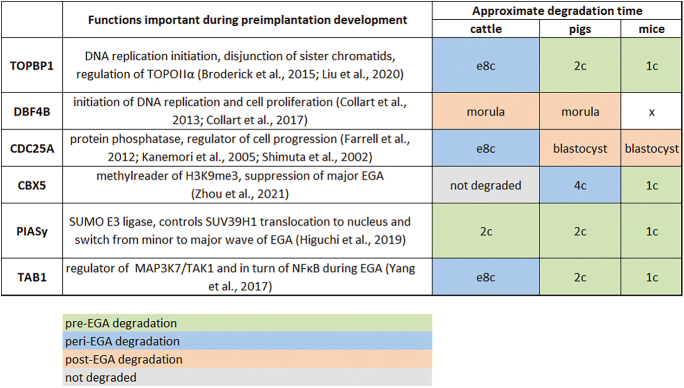
Functions of selected proteins and their approximate degradation timing during preimplantation development in cattle, pigs and mice. *X* = protein DBF4B not detected in mice; 1c, zygote; 2c, 2-cell stage embryo; 4c, 4-cell stage embryo; e8c, early 8-cell stage embryo.

For all the monitored genes except *CBX5*, there was a significant decrease in mRNA level at e8c or earlier (Figs 1A and 6A). Nevertheless, protein degradation occurred only in some proteins and seemed to be highly species-specific. The degradation of all the studied proteins is necessary for the progress of EGA in mice/Xenopus and occurs before EGA/MBT in these species. Such degradation timing was found in three of six studied proteins (TAB1, TOPBP1, CDC25A) in cattle (Fig. 9). TAB1, TOPBP1 and CDC25A are all involved in the regulation of cell cycle progression, and their degradation is thus likely related to the decrease in speed of division after EGA. In mice and pigs, the protein degradation profile of these proteins correlated in terms of the developmental stage (Fig. 7A, C, F, G), and occurred shortly after fertilization (TAB1, TOPBP1) or long after EGA (CDC25A). As some proportion of both TAB1 and TOPBP1 was still present throughout preimplantation development, we suppose that a small amount of the protein needs to be preserved to control cell cycle regulation, while the rest of the protein needs to be degraded. The function of TAB1 is largely regulated by several posttranslational modifications, especially ubiquitylation, phosphorylation and SUMOylation, which affect the function of the protein and can be seen as bands migrating at different molecular weights. Moreover, the localization of TAB1, TOPBP1 and CDC25A was both nuclear and cytoplasmic (Figs 1C, 3C, 6E). While the cytoplasmic localisation of TAB1 is common, cytoplasmic localisation of TOPBP1 and CDC25A is typical for cancer cells (Going *et al.* 2007, Morris *et al.* 2008, Forma *et al.* 2012, Al-Matouq *et al.* 2017). As the cytoplasmic localisation of otherwise nuclear proteins was relatively common in bovine embryos, we verified the specificity of the localization using bovine fibroblasts (Fig. S5). CDC25A is a highly posttranslationally modified protein, and Western blot analysis using anti-CDC25A antibodies usually shows more than one band. The fastest electrophoretic band of CDC25A is for the unphosphorylated, deubiquitylated form of CDC25A (Zhao *et al.* 2002). In bovine preimplantation embryos, we detected a CDC25A double band, with the fastest migrating band found in both 8c stages (Fig. 3B). This fastest band was, however, very weak, and thus we hypothesize that it was for a small remainder of unmodified protein that needs to be preserved for cell cycle control, similarly to TAB1 and TOPBP1. This is also indicated by the localization of CDC25A protein. At the earliest developmental stages, the protein was uniformly localized to both cytoplasm and nucleus. Later, the intensity of nuclear staining progressively increased, and at the blastocyst stage it became mostly nuclear. This clearly indicates the need for a small amount of CDC25A even in later stages after EGA. In pig and mouse embryos, we detected high levels of CDC25A protein until 8c, which is long after EGA, especially in mice. CDC25A degradation was found to be needed for the cell-cycle lengthening typical for the maternal-to-zygotic transition in *Drosophila*, *Xenopus*, zebrafish and mice (Kim *et al.* 1999, Dalle Nogare *et al.* 2009, Farrell *et al.* 2012, Knoblochova *et al.* 2023). Maintaining high levels of CDC25A during the later stages of development is thus highly surprising and warrants further investigation, especially in the context of its cell cycle-dependent degradation.

For the other types of degradation timing, always one protein corresponded to one timing (after fertilization – PIASY; after EGA – DBF4B; no degradation – CBX5). The degradation of PIASY shortly after fertilization is needed for the transition from the minor to major wave of EGA in mice (Higuchi *et al.* 2019, Yan *et al.* 2019), and we detected similar trends during early preimplantation development in both cattle and pigs (Fig. 7E). Similarly to the above-mentioned proteins, the protein was localized mostly to the cytoplasm in MII oocytes. After fertilization, the localization of PIASY changed to almost exclusively nuclear, which likely corresponds to the existence of an active and an inactive form of PIASY, of which the active form is present in the nucleus (Higuchi *et al.* 2019).

The most surprising results were found in CBX5 protein. CBX5, similarly to PIASY, had been found to be degraded during EGA in mice (Zhou *et al.* 2021). Nevertheless, the level of detected protein was the highest at both the early and late 8c stage, and CBX5 was the only protein in which no significant decrease in mRNA level was found. CBX5 is typically localized to chromocentres (De Koning *et al.* 2009). The formation of chromocentres correlates with EGA (Martin *et al.* 2006). While in mouse embryos CBX5 immunofluorescent staining becomes very weak after EGA, with no specific nuclear localization (Zhou *et al.* 2021), in bovine embryos CBX5 formed typical chromocentre clusters from e8c onwards (Fig. 4C). Thus, we suppose that CBX5 plays an important role in maintaining chromocentres during bovine preimplantation development, while this process is ensured by other protein(s) in mouse embryos. The CBX5 protein exhibited the greatest interspecies differences in detected molecular weight, likely caused by varying levels of phosphorylation. CBX5 is a highly phosphorylated protein, and its phosphorylation results in significant differences in band migration rate (Nishibuchi *et al.* 2019). Phosphorylation of CBX5 plays a significant role, among others, in nucleosome/chromatin binding or mitotic progression (Hiragami-Hamada *et al.* 2011, Chakraborty *et al.* 2014, Nishibuchi *et al.* 2014), and the interspecies differences in molecular weight are therefore likely related to the variability in its expression among species and, consequently, in its function during preimplantation development. The heterochromatin loosening important for EGA initiation is likely ensured by a decrease in PIAY levels in bovine embryos, as CBX5 collaborates with PIASY in maintaining of chromosomes in a heterochromatin state by sustaining H3K9 trimethylation (van Wijnen *et al.* 2021). PIASY has E3 SUMO ligase activity and promotes the SUMOylation-dependent targeting of CBX5 onto pericentric heterochromatin independently of H3K9me3. Interestingly, the utilization of SUMOylation seems to be one of the most interspecies-conserved PTMs (Maldonado-Saldivia *et al.* 2007, Kang *et al.* 2015, Huang *et al.* 2017, da Silva *et al.* 2022, Fang *et al.* 2024). Nevertheless, the mRNA expression of this pathway shows species-specific differences, especially in mice (Fig. 5; Higuchi *et al.* 2019, da Silva *et al.* 2022), which suggests some interspecies variability.

The DBF4B protein belongs to the last type of protein degradation timing. DBF4B was selected for the study as one of the replication partners of TOPBP1 homologue in Xenopus (Collart *et al.* 2013). We chose DBF4B, since it undergoes an interesting switch between its two variants Drf1 and DBF4 during the MBT/EGA in *Xenopus, Drosophila* and humans (Montagnoli *et al.* 2002, Collart *et al.* 2013, 2017, Torres-Zelada *et al.* 2022). The *Xenopus* Drf1 is degraded shortly after MBT, while Dbf4 is expressed not only during embryonic development, but also in somatic cells (Takahashi & Walter 2005, Silva *et al.* 2006, Collart *et al.* 2017). A similar expression profile was also found in the *Drosophila* variants (Chiffon A and B) (Torres-Zelada *et al.* 2022). The main role of DBF4 proteins is the initiation of DNA replication, so its degradation before MBT is needed to decrease the number of replication cycles and, consequently, for lengthening the cell cycle (Collart *et al.* 2017). Nevertheless, the level of DBF4B protein was stable up to L8c in both cattle and pigs, with a decrease at the morula and blastocyst stage, which could be related to a lesser extent of slowdown of cell division in mammals. On the other hand, the mRNA level rapidly decreased after the 4-cell stage in cattle, i.e. before EGA. This suggests that there might also be a second variant of DBF4B in cattle. Nevertheless, we have not detected such a variant. The occurrence of such a switch, however, seems to be a common process during EGA, as a switch in protein variant expression also occurs in Cullin1 during bovine EGA and CDC6 in *Xenopus* embryos (Tikhmyanova & Coleman 2003, Kepkova *et al.* 2011). In mouse oocytes and embryos, the DBF4B protein was undetectable. As suggested by the protein function, DBF4B/Drf1 should be a nuclear protein (Montagnoli *et al.* 2002). Nevertheless, similar to other proteins involved in this study, the protein was distributed throughout both the cytoplasm and nuclei in bovine embryos, and was even absent from nuclei in 2c embryos and blastocysts. This suggests that DBF4B might be assigned for degradation and is not needed at this stage.

In summary, the same timing of degradation across all three species was very rare among the analysed proteins. It occurred only in the case of PIASY. For several proteins, the developmental stage at which degradation occurred matched between mouse and pig, while in mouse and cattle, degradation occurred at the same developmental phase with respect to the timing of EGA (TOPBP1, CDC25A, TAB1). In cattle, no decrease in CBX5 protein levels was observed. The DBF4B protein is not present in mouse, but in pig and cattle it shows a similar expression profile.

As UPS is traditionally considered the most important regulator of protein degradation during preimplantation development, PTMs such as ubiquitination are supposed to play a crucial role during early embryogenesis. The utilization of PTMs also likely contributes to the species-specific differences in protein processing. The mechanisms by which PTMs are utilized are evolutionarily well conserved, with divergence primarily in the extent and temporal dynamics of their regulation (Beltrao *et al.* 2013, Schönichen *et al.* 2013). A typical feature is the species-specific potential of mutual compensation among PTM-regulating pathways. For example, there is a functional redundancy between p38 MAPK and ERK1/2 pathways in bovine but not in murine preimplantation embryos, or differential regulation of the Hippo pathway between these two species (Madan *et al.* 2005, Sharma *et al.* 2021). The different temporal profile of protein degradation might be further related to differences in mRNA expression of genes involved in UPS and SUMOylation pathways (e.g. *NEDD8*, *SUMO1*, *UBAP1*) in rhesus monkey, mouse and cattle (Mtango & Latham 2007, Benesova *et al.* 2016). Interestingly, demethylation, regulating, among other things, transcription level, is conserved between mouse and human, even though their timing of EGA is markedly different (Ross & Sampaio 2018). Nevertheless, the utilization of PTMs in mammalian species other than mice and humans is still largely unexplored.

In conclusion, we have found that maternal protein degradation during mammalian preimplantation development is species-specific. The timing of degradation differs even in protein homologues with high sequence homology, and the degree of similarity in the timing of degradation does not correspond to the evolutionary relatedness between species. The need for mRNA expression of most of the studied genes dramatically decreased before EGA. However, the protein expression level was also decreased in only some of these genes (*TAB1*, *TOPBP1*, *CDC25A*, *PIASY*). For many proteins, the protein localization in embryos tended to be cytoplasmic, even though nuclear localization is typical for other cell types. Cytoplasmic localization likely reflects the need to store protein reserves in an inactive form. The way in which maternal proteins are targeted for degradation remains to be elucidated, and this study provides the basis for such work.

## Supplementary materials



## Declaration of interest

The authors declare that there is no conflict of interest that could be perceived as prejudicing the impartiality of the work reported.

## Funding

This work was primarily supported by a project grant from the Czech Science Foundation (GAČR; 23-05108S). ARB was also supported by the Ministry of Education, Youth and Sports of the Czech Republic, Operational Programme Research, Development and Education, the project ‘EXCELLENCE in molecular aspects of the early development of vertebrates’ (Ministerstvo školství, mládeže a tělovýchovy České republiky: CZ.02.1.01/0.0/0.0/15_003/0000460) and by the Czech Academy of Sciences (PPLZ; L200452402).

## Author contribution statement

Bovine embryo culture was performed by VK and JK. Porcine embryo culture was carried out by ARB and RP. Murine embryo culture was conducted by AS. Experiments were performed by VK, TT, and SA. Data analysis was conducted by TT and VK. Manuscript writing was done by TT and VK. Manuscript reading and correction were carried out by VK, SA, JK, RP, ARB, AS, and TT. Funding was provided by TT, RP, and ARB.

## Data availability

The data underlying this article will be shared on reasonable request to the corresponding author.
