# Supplementary material for: The timing of maternal protein degradation during mammalian preimplantation development is species-specific
Source: Reproduction. 2025 Aug 14;170(3):e250007. doi: 10.1530/REP-25-0007 (PMC12358800; doi:10.1530/REP-25-0007)

S Box 1

The terminology of processes during early embryogenesis

During early embryogenesis, the control of the development of the embryo has to be switched from oocyte to embryo itself. The nomenclature used for this process and its parts is not uniform neither between species nor between research groups. The whole process is usually termed maternal-to-zygotic transition (MZT). Nevertheless, the most important part of this transformation, the activation of embryonic transcription, is laso referred to as such by some researchers. More often, the terms zygotic genome activation (ZGA) for murine embryo; embryonic genome activation (EGA) for bovine or porcine embryo and midblastula transition (MBT) for non-mammalian species is used. This is connected not only to the fact that activation of transcription occurs shortly after fertilization in mouse, but also to the different use of the term zygote. Most often, it is used for 1-cell embryo, but some researchers use this term even for later preimplantation stages. In this paper, we will use the term **maternal-to-zygotic transition** for the whole process including not only activation of transcription, but also several other processes from activation of the oocyte to the depletion of maternal products (Marlow, 2010). For the activation of embryonic transcription in mammalian species the term **embryonic genome activation** will be used, for the same process in lower vertebrates and non-vertebrate species, the term **midblastula transition** will be used.

Materials and methods

EGA timing in mice

Embryos collected in different time periods after isolation (zygote) until the 2c stage were used for detection of timing of EGA. Fixed embryos were incubated in 0.5 % (v/v) TritonX-100 for 15 min and subsequently washed by 0.1% PVA in PBS. Unless otherwise stated, all subsequent steps were done in PBS supplemented with 0.1 % PVA. Embryos were blocked with 0.2% BSA in PBS for 1 h and incubated with anti-phospho RNA Pol II (Ser2) (MABE953, Millipore) primary antibody overnight at 4 °C. After washing, the embryos were incubated with Goat anti- Rat IgG (H+L) Cross-Adsorbed Secondary Antibody, Alexa Fluor™ 647 (Invitrogen) for 1h at room temperature darkling. After washing, the nuclei were stained and embryos were mounted in SlowFade™ Diamond Antifade Mountant with DAPI (4',6-Diamidine-2'-phenylindole dihydrochloride, Invitrogen). The samples were examined using Leica TCS SP5 (Leica Microsystems AG, Wetzlar, Germany). The images were processed using Image J software.

Fibroblast culture

Bovine fibroblasts were thawed and culture in D-mem (Dulbecco's modified eagle's) medium supplemented with 10% FBS. After two days, cells were passaging and for PCR or western blot, cell were washed with PBS and frozen. For immunofluorescence, fibroblasts were culture on pre-prepared slides coated with gelatin and subsequently fixed with 4% PFA and stored in PBS at 4 °C.

Fibroblast immunofluorescence staining – antibody specificity

Cells were incubated in 0.5 % (v/v) TritonX-100 for 15 min. All subsequent steps were done in PBS supplemented with 0.3 % (w/v) BSA and 0.05 % (w/v) saponin (PBS/BSA/sap). Cells were blocked with 2 % normal goat serum (NGS) or PBS supplemented with 5 % BSA for 1 h and incubated with appropriate primary antibody (see Table S2) in PBS/BSA/sap overnight at 4 °C and subsequently for 1 h with appropriate secondary antibody (Table S2) in PBA/BSA/sap. Afterwards, the nuclei were stained and embryos were mounted in SlowFade™ Diamond Antifade Mountant with DAPI (4',6-Diamidine-2'-phenylindole dihydrochloride, Invitrogen, Thermo Fisher Scientific). Controls of immunostaining specificity were carried out by omitting the primary antibody.

The samples were examined using Leica TCS SP5 (Leica Microsystems AG). The images were processed using Image J software.

Tables

Table S1 Antibodies

	Company	Catalog No.	Working dilution (WB)	Blocking solution (WB)	Working dilution (IF)	Blocking solution (IF)
CBX5	Abcam	Ab77256	1:1000	Azure	1:500	5% BSA
CDC25A	ThermoFisher Scientific	PA5-77902	1:1000	Azure	1:100	2% NGS
DBF4B	ThermoFisher Scientific	PA5-71078	1:1000	Azure	1:100	2% NGS
GAPDH	Merck Sigma- Aldrich	G9545	1:40000	Azure	x	x
PIASy	Merck Sigma- Aldrich	SAB4502145	1:1000	Azure	1:100	2% NGS
TAB1	ThermoFisher Scientific	MA5-32953	1:500	3% BSA	1:50	2% NGS
TOPBP1	ThermoFisher Scientific	PA5-78356	1:500	Azure	1:100	2% NGS
Ubiquitin	Cell Signaling Technology	3936	x	x	x	x

Table S2 Primer details

Primer	Sequences	Annealing temperature (°C)	Amplicon size (bp)
Cbx5	F: GAGACGTTAGCGTGAGTGGT<br>R: CCTTAACCACACGCCTGTCT	55	271
Cdc25A	F: ATGGGCTCCTCCGAATCAAC<br>R: AGAGCTTCTGAGGTAGGGAA	59	118
Dbf4b	F: TGGTTGCTCATGGAAAACGG<br>R: CAGCCACCAATTTCTTGCCC	62	108
H3f3A (van der Weijden et al., 2017)	F: ACTGGCTACAAAAGCCGCTC<br>R: ACTTGCCTCCTGCAAAGCAC	55	232
Piasy	F: GTGCAAGGCACTGGTCAAAG<br>R: GGTGGGCTTCTTCTCGTTCA	59	193
Tab1	F: CCGATCATTGCAGAGCCTGA<br>R: GTAGCCAAAGTTCCGCACCA	62	309
TopBP1	F: TCGTACTCTTTCAGATGTCAGCC<br>R: AGGTGCCTGAAATGCACTGA	62	142

References

Marlow F L 2010 Maternal Control of Development in Vertebrates: My Mother Made Me Do It!. San Rafael (CA): Morgan & Claypool Life Sciences (https://doi.org/10.4199/C00023ED1V01Y201012DEB005)

van der Weijden Vera A, Shuai C, Bauersachs S, *et al*. 2017 Gene expression of bovine embryos developing at the air-liquid interface on oviductal epithelial cells (ALI-BOEC). *Reprod Biol Endocrinol* **15** 29178958. (https://doi.org/10.1186/s12958-017-0310-1

Results

Figure S1

Determination of EGA initiation in mice

A positive fluorescent signal in the nucleus was detected in all zygotes 28 hours post hCG injection and subsequently also in all 2-cell stage using the anti- phospho RNA Pol II antibody. Thus, mice zygotes were collected soon after isolation and were considered as pre-EGA stage and 2c stage embryos as EGA/post-EGA stage.
Scale bars: 20 µm, DNA (DAPI): blue, phosphorylated RNA polymerase II: red.

Pre-EGA (zygotes) EGA-stage (late zygotes and 2c)

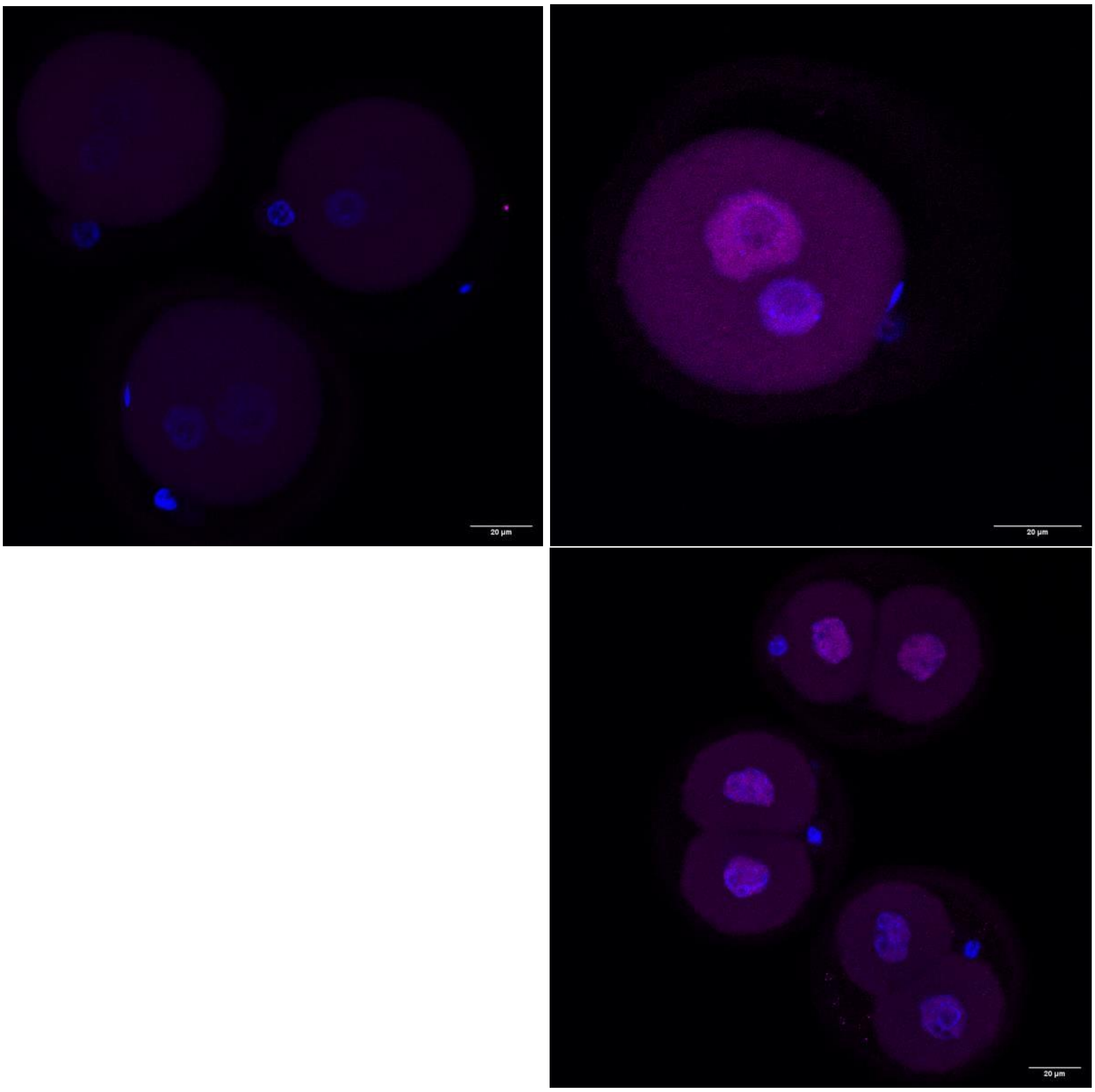

Figure S2

Homology of selected proteins between species. Multiple sequence alignment of proteins A) TOPBP1, B) DBF4B, C) CDC25A, D) CBX5, E) PIASy and F) TAB1 from cattle, pig, mouse and human. Sequences were retrieved from NCBI Protein database (accessed May 2025; https://www.ncbi.nlm.nih.gov/protein) and aligned using Clustal Omega (https://www.ebi.ac.uk/jdispatcher/msa/clustalo). Visualization and annotation were performed in Jalview (2.11.4.1; https://www.jalview.org/). Colored residues represent sequence similarity and antibody epitopes (according to the human sequence) are marked. Conservation, quality, consensus and occupancy scores are shown below the alignment. Conservation = degree of amino acid conservation across species (max = 10), quality = average similarity of each column based on substitution matrix, consensus= most frequent amino acid at each position, occupancy = proportion of sequences with a non-gap residue at each position. A) Multiple sequence alignment of TOPBP1 from selected species. The epitope recognized by the antibody is located within the central region of the human TOPBP1 protein based on manufacturer's description (approx. residues 700 – 800). The exact sequence of immunogen is proprietary and not disclosed. Due to the large size of the TOPBP1 protein, we chose to present a partial sequence alignment (amino acids 450–1000), focusing on the central region relevant for this study. B), C) and F) Multiple sequence alignment of DBF4B, CDC25A and TAB1 from selected species. The epitopes recognized by the antibodies are directed towards the N-terminal regions of human DBF4B, CDC25A or TAB1, however the exact sequences of immunogen are proprietary and not disclosed. The N-terminal regions are marked in the alignments. D) Alignment of amino acid sequence of protein CBX5. The antibody was raised against synthetic peptide within the region spanning amino acids 50-150 of human CBX5. This region is marked in the alignment. The exact immunogen sequence is proprietary and not disclosed. E) Multiple sequence alignment of PIASy from selected species. The antibody was raised against a region within amino acids 451 – 500 of the human PIASy sequence (immunogen range), this region is marked in the alignment.

For better understanding, the similarity between selected proteins are described in following table. Pairwise amino acid sequence identities between species were calculated using Protein BLAST (BLASTp) at the NCBI server (https://blast.ncbi.nlm.nih.gov/Blast.cgi). For each protein, the bovine sequence was used as the query, and sequences from other species were aligned to evaluate identity percentages.

**cattle**	**mouse**		**pig**	
	**ID**	**Homology with bovine protein (%)**	**ID**	**Homology with bovine protein (%)**
**DBF4B** (XP_059734544.1)	x	x	XP_020922518.1	78.84
**TOPBP1** (NP_001193695.1)	XP_006511769.3	73.87	XP_020925124.1	92.51
**CDC25A** (NP_001094570.1)	NP_031684.3	83.3	XP_020937595.1	95.25
**CBX5** (XP_059742588.1)	NP_001345879.1	97.91	XP_020947403.1	100
**PIASy** (NP_001076951.1)	NP_067476.2	86.82	XP_003354055.1	97.86
**TAB1** (XP_024847542.1)	NP_079885.2	96.77	XP_020946588.1	95.78

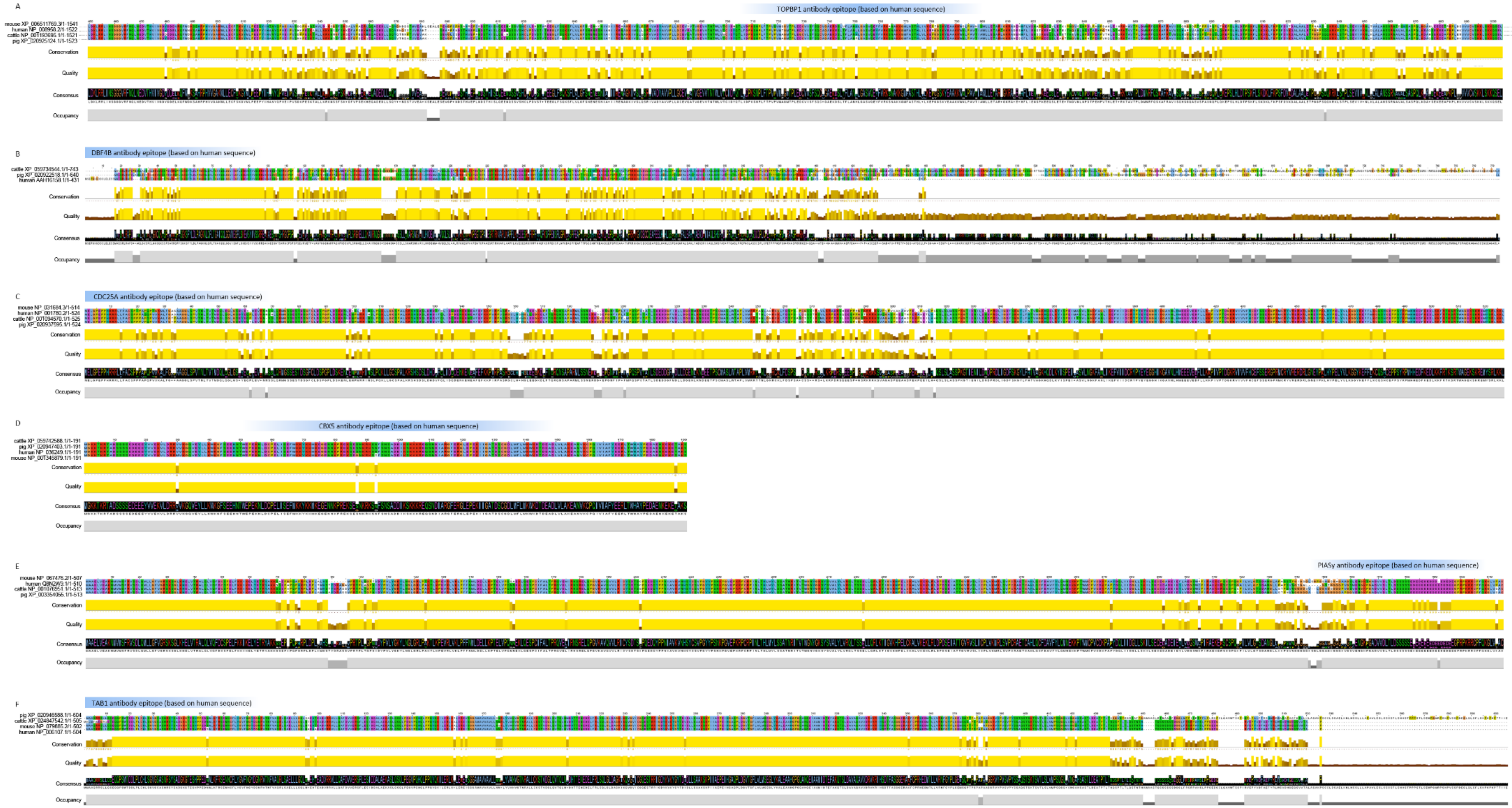

A
TOPBP1 antibody epitope (based on human sequence)
mouse XP_006511769.3/1-1541
human NP_008958.2/1-1522
cattle NP_001193695.1/1-1521
pig XP_020925124.1/1-1523
Conservation
Quality
Consensus
Occupancy
B
DBF4B antibody epitope (based on human sequence)
cattle XP_059734544.1/1-743
pig XP_020922518.1/1-640
human AAH16158.1/1-431
Conservation
Quality
Consensus
Occupancy
C
CDC25A antibody epitope (based on human sequence)
mouse NP_031684.3/1-514
human NP_001780.2/1-524
cattle NP_001094570.1/1-525
pig XP_020937595.1/1-524
Conservation
Quality
Consensus
Occupancy
D
CBX5 antibody epitope (based on human sequence)
cattle XP_059742588.1/1-191
pig XP_020947403.1/1-191
human NP_036249.1/1-191
mouse NP_001345879.1/1-191
Conservation
Quality
Consensus
Occupancy
E
PIASy antibody epitope (based on human sequence)
mouse NP_067476.2/1-507
human Q8N2W9.1/1-510
cattle NP_001076951.1/1-513
pig XP_003354055.1/1-513
Conservation
Quality
Consensus
Occupancy
F
TAB1 antibody epitope (based on human sequence)
pig XP_020946588.1/1-604
cattle XP_024847542.1/1-505
mouse NP_079885.2/1-502
human NP_006107.1/1-504
Conservation
Quality
Consensus
Occupancy

Figure S3

Expression of CBX5 protein in bovine, pig and mouse GV oocytes

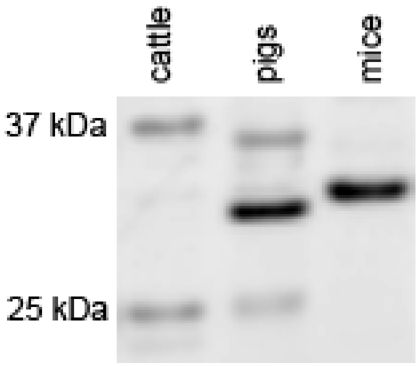

Figure S4

Expression of TAB1 protein in bovine, pig and mouse GV oocytes.

The bands marked with an arrow were used in Figure 8 in individual species.

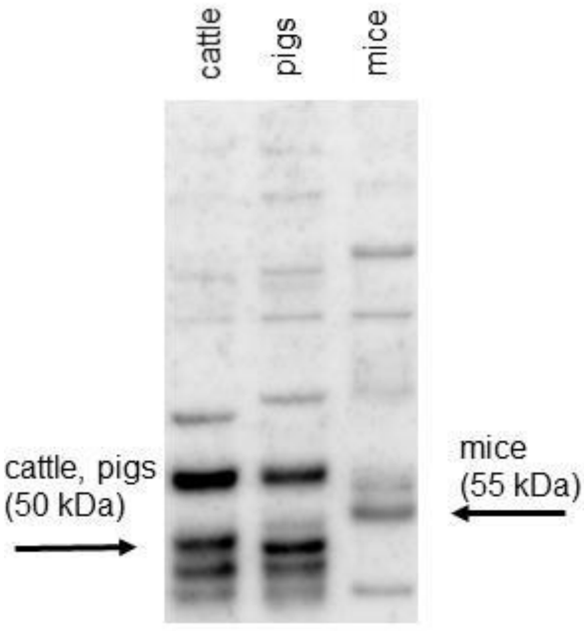

Figure S5

Localization of DBF4B, TOPBP1, CDC25A, CBX5, PIASy and TAB1 protein (confocal laser scanning microscopy) in bovine fibroblasts. Representative image of each protein is shown. Scale bars: 20 µm, DNA (DAPI): blue, protein: red.

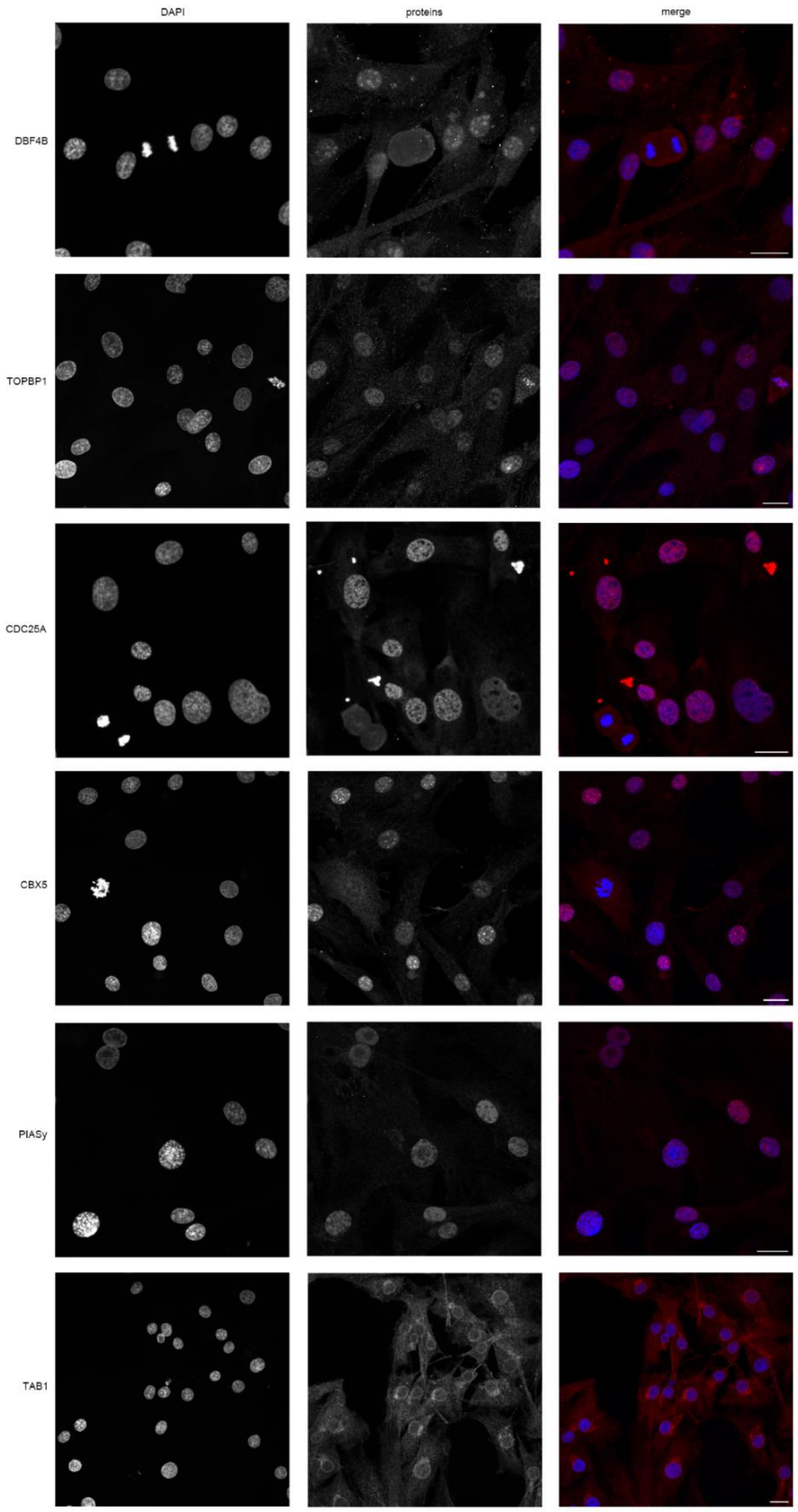

Supplement: Supplementary file 1 [file supplementary_materials.pdf]
